# Differences in dual-task performance and prefrontal cortex activation between younger and older adults

**DOI:** 10.1186/1471-2202-14-10

**Published:** 2013-01-18

**Authors:** Hironori Ohsugi, Shohei Ohgi, Kenta Shigemori, Eric B Schneider

**Affiliations:** 1 Graduate School of Health Sciences, Seirei Christopher University, 3453 Mikatahara-Cho, Hamamatsu-City, Shizuoka, 433-8558, Japan; 2 Department of healthcare, Kansai University of Health and welfare science, 3-11-1, Asahigaoka, Kashiwara-City, Osaka, 582-0026, Japan; 3 Johns Hopkins School of Medicine, Center for Surgical Trials and Outcomes Research, 600 N. Wolfe St, Baltimore, MD, 21205, USA

**Keywords:** Dual-task, Near-infrared spectroscopy, Executive function, Attentional function

## Abstract

**Background:**

The purpose of this study was to examine task-related changes in prefrontal cortex (PFC) activity during a dual-task in both healthy young and older adults and compare patterns of activation between the age groups. We also sought to determine whether brain activation during a dual-task relates to executive/attentional function and how measured factors associated with both of these functions vary between older and younger adults.

**Results:**

Thirty-five healthy volunteers (20 young and 15 elderly) participated in this study. Near-infrared spectroscopy (NIRS) was employed to measure PFC activation during a single-task (performing calculations or stepping) and dual-task (performing both single-tasks at once). Cognitive function was assessed in the older patients with the Trail-making test part B (TMT-B). Major outcomes were task performance, brain activation during task (oxygenated haemoglobin: Oxy-Hb) measured by NIRS, and TMT-B score. Mixed ANOVAs were used to compare task factors and age groups in task performance. Mixed ANOVAs also compared task factors, age group and time factors in task-induced changes in measured Oxy-Hb. Among the older participants, correlations between the TMT-B score and Oxy-Hb values measured in each single-task and in the dual-task were examined using a Pearson correlation coefficient.

Oxy-Hb values were significantly increased in both the calculation task and the dual-task within patients in both age groups. However, the Oxy-Hb values associated with there were higher in the older group during the post-task period for the dual-task. Also, there were significant negative correlations between both task-performance accuracy and Oxy-Hb values during the dual-task and participant TMT-B scores.

**Conclusions:**

Older adults demonstrated age-specific PFC activation in response to dual-task challenge. There was also a significant negative correlation between PFC activation during dual-task and executive/attentional function. These findings suggest that the high cognitive load induced by dual-task activity generates increased PFC activity in older adults. However, this relationship appeared to be strongest in participants with better baseline attention and executive functions.

## Background

Cognitive functions, such as selective attention, memory and executive function, decline with age [1,2]. The population is aging with ever larger numbers of individuals reaching ages where decline in function is more common [3]. Age-related changes to the brain have been shown to occur earliest in the prefrontal cortex (PFC) [4]. The PFC has been associated with memory, attention, executive function and emotion, as well as playing a role in a variety of other complex cognitive functions [5-7]. Pathological or age-related changes in this area have been associated with a number of neuropsychiatric disorders with cognitive, emotional, behavioral, or affective manifestations. These disorders include schizophrenia, depression and dementia [8]. The decline in cognitive ability associated with aging has been related to disuse of certain cognitive functions and a corresponding reduction in brain activity [9]. Previous studies have suggested that increasing activity in the PFC might be able to prevent, or moderate, age-related changes to the brain [10]. Neuronal activation leads to increase of regional cerebral blood flow [11,12]. In addition to increased blood flow in active areas, other factors associated with the transport of certain blood-borne factors have been shown to be increased in brain areas in the presence of increased neural activity. For example, Nishijima, et al. [13] showed that neuronal activity elicited by electrical, sensory, or behavioral stimulation increases insulin-like growth factor-1 (IGF-I) input in activated regions. IGF-1 modulates neuronal growth, survival, neural excitability, and cognitive function. Therefore, it might be hypothesized that regular activation of the PFC may prevent decline of the brain functions associated with this most important brain area.

Numerous studies have reported that regular physical activity and cognitive training are associated with better cognitive function [14-18]. Therefore, it is thought that a combination of both cognitive and physical activity might be useful in preventing, or slowing, the decline of cognitive function seen in many older adults. There is growing evidence that dual-task (combining both physical and cognitive activity) training better supports healthy executive function, working memory and the ability to divide attention than either physical or cognitive training in isolation [19-23]. Current literature suggests that dual-task activity also may be an effective tool to prevent the decline of overall cognitive function [24-26]. Pedroso et al. [24] showed that, over time, dual-task training improves dual-task performance in patients with mild to moderate dementia. Erickson et al. [26] showed that improvements of dual-task performance correlate with measured changes in brain activation. Also several neuroimaging studies have revealed increased brain activity in the PFC during dual-task activity. D’Esposito et al. [27] compared single-task performance with a concurrent performance of both tasks and discovered that activation of the PFC occurred only during the dual-task condition whereas this area was not active during either single-task. Koechlin et al. [28] also demonstrated increased activation in PFC bilaterally during a dual-task condition compared to similar measurements when a single-task was being performed. However, Callicott et al. [29] have demonstrated an ‘inverted-U’ shaped neuro-physiological response as subjects are moved from activities producing the lowest cognitive load toward those producing the highest cognitive load leading these authors to suggest that excessive processing demands are actually accompanied by a diminution in cortical activity. In another study, Reuter-Lorenz and Cappell [30] suggested older adult demonstrated overactivation in PFC during a cognitive task.

A number of studies have suggested that older age is associated with substantial differences in hemodynamic response to stimuli. For example, Schroeter [31] suggested that aging decreases the hemodynamic response in the frontal association cortex during cognitive task. In this way, the brain activation during dual-task in older adults was still controversial. Therefore, the purposes of this study were as follows: 1) to clarify and compare task-related changes in PFC activity, measured by examining changes in brain blood oxygen levels comparing brain activation during single-task activities with a dual-task; 2) to examine age-related differences across single- and dual-task conditions between healthy young individuals and healthy older adults; 3) to examine whether levels of brain activation during dual-task activity are associated with baseline levels of executive and attentional functioning in the older age group.

## Methods

### Participants

**Figure 1:**
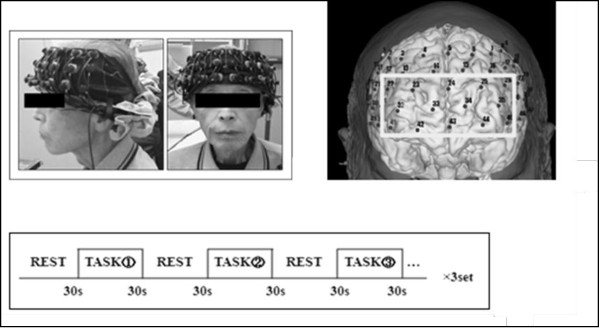
**NIRS probe setting and task protocol.** On the left: NIRS probe setting, on the right: brain mapping and ROI setup, bottom: task protocol.

Thirty-five healthy volunteers participated in this study. The younger group was selected from individuals aged 21–35 years of age and included 20 individuals. The older group was selected from individuals greater than 65 years of age and included 15 individuals. All participants were healthy and without impairment of visual or auditory senses. Both young and old participants demonstrated independence in activities of daily living. Informed consent was obtained from all participants prior to enrollment. Written informed consent was obtained from the patient for publication of the image in Figure 1. A copy of the written consent is available for review by the Editor-in-Chief of this journal. This study was approved by the Ethics Committee of Seirei Christopher University (approval No. 10054).

### Protocol

The independent motor execution task (physical single-task) was performed by seated participants stepping in place with each foot while counting out loud forward from zero. The cognitive single-task involved serial subtractions of 7 beginning with 100 and continuing as follows: 100, 93, 86, 79, etc. The results of each calculation were spoken out loud so accuracy could be assessed. Both the physical and cognitive single-tasks were performed in the seated position with eyes closed. The dual-task combined the cognitive and motor execution tasks with seated participants performing another series of serial 7 subtraction problems while stepping, again with their eyes closed. PFC activation was recorded using near-infrared spectroscopy (NIRS) during both the physical and cognitive single-task activities as well as during the dual-task. Figure 1 demonstrates the experimental protocol. Each subject was instructed to perform either one of the single-tasks or the dual-task randomly after a rest period. In order to randomize the order of tasks across individuals, subjects selected one task pattern from the six possible patterns by lot (the tasks and the associated patterns for selection were as follows: 1) stepping task, 2) calculation task, 3) dual-task, 1-2-3/ 1-3-2/ 2-1-3/ 2-3-1/ 3-1-2/ 3-2-1,) (Figure 1). Rest and task periods were set for 30 seconds and each task was repeated three times. During the rest period, participants were asked to simply count out loud at their own pace.

### Measurement of brain activation

PFC activity was assessed using the NIRS (ETG-7100, Hitachi Medical, Tokyo, Japan) which uses two wavelengths of near-infrared light (695 nm and 830 nm). The distances between the injectors and detectors were 3.0 cm, and it was determined that the machine measures points associated with the surface of the cerebral cortices [32,33]. The NIRS probes contain 3 × 10 lines of plastic transmitter/receiver shells (47 channels). We placed the optode grid on the head of the subjects according to 10–20 positions and the lowest probes were placed along Fp1-Fp2 (Figure 1). NIRS measured the relative changes in the concentrations of oxygenated hemoglobin (Oxy-Hb), deoxygenated hemoglobin (Deoxy-Hb) and total hemoglobin (Total-Hb). Fundamental data of Hb was display and uses [mM*mm], which is an index of Hb change as a scaled variable. We used changes in oxy-Hb values as indicators of change in the regional cerebral blood volume, because the oxy-Hb is more sensitive than deoxy-Hb as a parameter for measuring the blood flow changes associated with brain activation [34]. Sampling frequency was 10 Hz, the processed moving average was 5 seconds, a 0.5 Hz low-pass filter was used to remove the effects of Mayer waves and a 0.01 Hz high-pass filter was used to remove baseline drift. Baseline corrections were made using the least squares method based upon the value of the measurement 10 seconds before task initiation and the value of the measurement 20–30 seconds after task completion. The task was repeated three times and the data from each of the three task blocks were averaged. These analyses were performed using the ‘integral mode’ of the ETG-7100 software. The Oxy-Hb data produced by the NIRS probes were segregated by the region of interest (ROI) [35], and the probes focused on the PFC were isolated for analysis. 14 channels (probe number 22, 23, 24, 25, 26, 32, 33, 34, 35, 41, 42, 43, 44, 45) corresponded to the PFC and were selected as the ROI (Figure 1). Average values for Oxy-Hb were calculated across the whole task period to confirm the task related change, and at each 10 second period to consider the time-dependent change, namely, pre-task, task 0-10s, task 10-20s, task 20-30s, post task 0-10s, post task 10-20s, post task 20-30s.

### Task performance

The number of steps made by each participant during both the stepping single-task and the combined dual-task were counted. The total number of serial 7 subtractions performed were counted and the numbers of correct and incorrect subtractions were recorded during both the subtraction single-task and the combined subtraction/stepping dual-task. The proportion of correct subtractions was calculated for both single and dual-task trials.

### Measurement of baseline cognitive function among the older adults

The Mini-Mental State Examination (MMSE) and Trail-Making Test (TMT) were performed for assessment of cognitive function in older participants. MMSE is one of the most common tools to screen for cognitive impairment in older adults [36]. The MMSE screens for impairment in overall brain function (i.e. the frontal, parietal, and temporal lobes) [37]. The TMT assesses several cognitive functions including working memory, attention, motor speed and more [38,39]. The TMT consists of two parts, A and B. Performance on TMT Part B (TMT-B) has been associated with PFC-related cognitive functions, including executive function, attention and working memory [39,40]. Because we were specifically examining PFC activity and function and wanted to determine if there was a relationship between baseline cognitive functioning and PFC activity in the older adult group, TMT-B was administered to the older participants. TMT-B consists of numbers from 1 to 13 and Japanese characters scattered on a sheet of paper, and participants must draw a line alternately between consecutive numbers and the appropriate sequential Japanese character. The time required to complete the TMT-B task was measured for each participant and was collected as required, accounting for the number of errors present. The number of errors in TMT has been associated with attentional control, working memory and executive functioning [41]. Errors involve drawing a line between a particular character or number and an inappropriate number, or failing to draw a line between characters in accordance with the instructions.

### Statistical analysis

To compare task factors and age groups in task performance (number of steps, total number answered, proportion of calculations performed correctly), we performed mixed ANOVAs respectively with group (younger vs. older) as the between subjects variable and task (single vs. dual) as the within-subjects variable. To compare the Oxy-Hb values associated with task factors and age groups, we performed mixed ANOVA with age group (younger vs. older) as the between subjects variable and task (stepping vs. calculate vs. dual) as the within-subjects variable. To compare task factor, age group and time factor in task-induced changes in Oxy-Hb values at various times across the study period using the longitudinally collected NIRS data, we performed mixed ANOVA with group (younger vs. older) as the between subjects variable and, task (stepping vs. calculate vs. dual) and time factor (pre-task, task 0-10s, task 10-20s, task 20-30s, post task 0-10s, post task 10-20s, post task 20-30s) as the within-subjects variables. Post hoc tests were performed using the Bonferroni method to reduce the possibility of Type I errors [42]. To examine the relationship between cognitive functioning and brain activation, the correlation between the TMT-B score (number of errors), Oxy-Hb values in whole task period in each task and dual-task performance in the older age group was analyzed using a Pearson correlation coefficient. All statistical analyses were performed using IBM SPSS Statistics 19 (SPSS, Inc., an IBM Company, Tokyo, Japan), the level of statistical significance was set at 0.05.

## Results

### Background of participants

The younger participants (10 males and 10 females) ranged in age from 22 to 35 years (mean: 26.0, S.D. 3.6) and the older participants (6 males and 9 females) ranged in age from 69 to 87 years (mean: 77.9, S.D. 5.3). None of the older participants had a score below 25 on the MMSE (mean MMSE score: 28.9, S.D. 1.5). The mean time required for the older participants to complete the TMT-B was 161.4 seconds (S.D. 105.4) and the mean number of error in TMT-B was 2.8 (S.D. 2.6). The MMSE and TMT were not given to the younger participants.

### Task performances

There were main effects of age group on the proportion of calculations performed correctly (F _(1, 33)_ = 10.02, *P* < 0.01, partial η^2^ = 0.23) and task factor in number of steps (F _(1, 33)_ = 7.39, *P* = 0.01, partial η^2^ = 0.18). There were no main effects of task factor in proportion performance correctly (F _(1, 33)_ = 0.04, *P* = 0.84), in total number answered (F _(1, 33)_ = 0.04, *P* = 0.84). Also, no main effect was shown for age group in total number answered (F _(1, 33)_ = 0.11, *P* = 0.74) and in number of steps (F _(1, 33)_ = 0.56, *P* = 0.46). The age group by task factor interaction was not significant in each parameter (in number of steps (F _(1, 33)_ = 0.28, *P* = 0.10), in total number answered (F _(1, 33)_ = 0.00, *P* = 0.97), in proportion performance correctly (F _(1, 33)_ = 0.45, *P* = 0.51)). However, the post hoc test showed that the number of steps was significantly lower for the dual-task compared with the single-task in the older group (*P* < 0.01). Older participants performed fewer computations correctly than younger participants in both the computational single-task (85.4% vs. 96.3%, respectively, *P* < 0.01) and the combined dual-task (87.5% vs. 96.0%, respectively, *P* < 0.01) (See: Table 1).

**Table 1:** Task performance

	**Younger group**		**Older group**	
	**Single-task**	**Dual-task**	**Single-task**	**Dual-task**
Number of steps ^a^	40.8 ±9.1	39.5 ± 9.7	45.5 ± 11.0	40.1 ± 14.0*
Total number answered ^b^	8.1	8.3	7.7	8.0
	(2.6-19.7)	(2.0-19.3)	(4.0-20.3)	(3.3-24.0)
Proportion performed correctly ^b^	96.3	96.0	85.4**	87.5**
	(72.9-100)	(71.4-100)	(41.7-100)	(52.9-100)

a: mean ± S.D., b: Median (range), *: significant different between single-task and dual-task (*P* < 0.05), **: significant different between younger and older group (*P* < 0.01).

The number of steps was significantly decreased in dual-task compared with single task in the older group. The proportions performed correctly were significantly lower in the older group than in the younger group in both tasks.

## NIRS results

**Figure 2:**
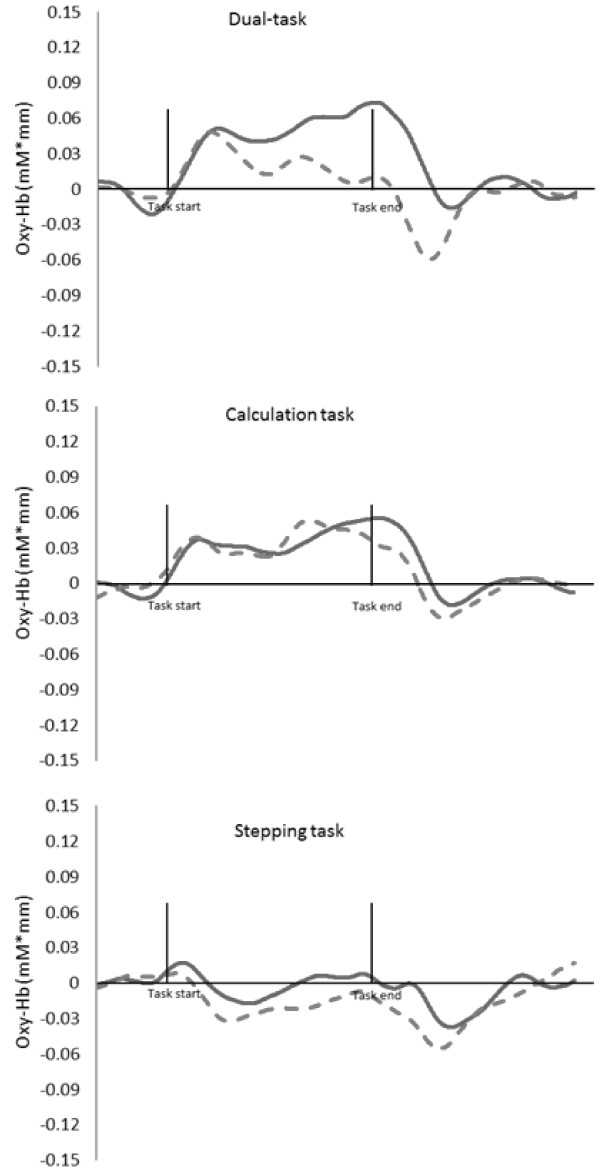
**Hemodynamic changes in PFC in each task (true time courses).** Top: in dual-task, middle: in calculation task, bottom: in stepping task, solid line: Older group, dotted line: Younger group.

**Figure 3:**
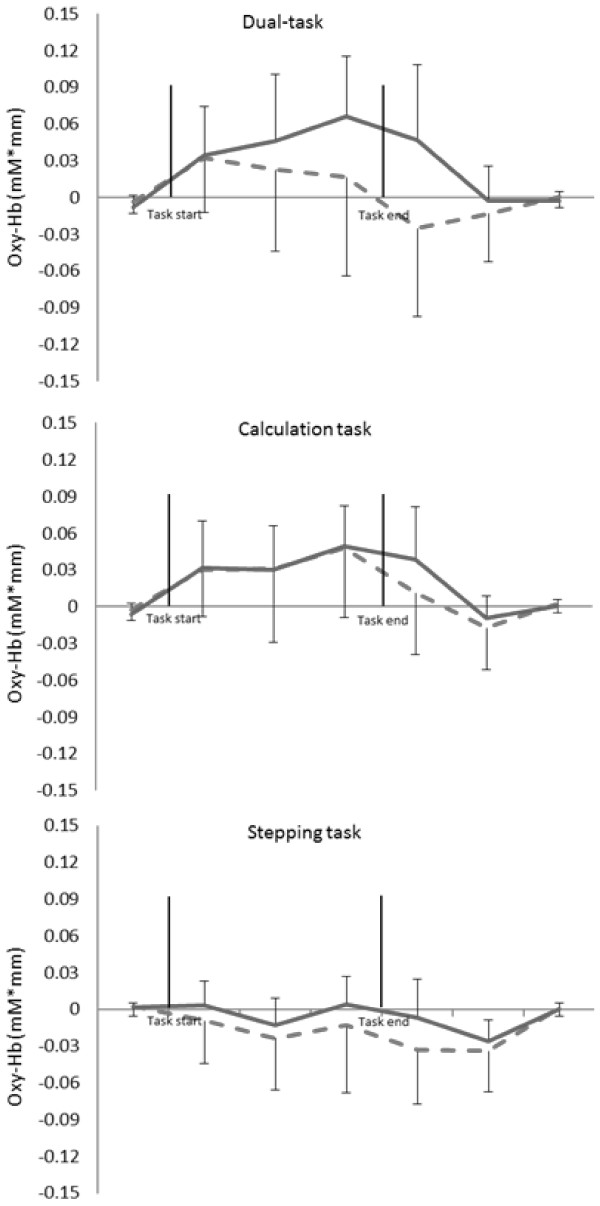
**Hemodynamic changes in PFC in each task (averaged in 10 sec).** Top: in dual-task, middle: in calculation task, bottom: in stepping task, solid line: Older group, dotted line: Younger group.

Table 2 showed the Oxy-Hb value in whole task period. The Oxy-Hb values across the entire testing period showed a significant relationship with task performance (F _(2, 32)_ = 23.57, *P* < 0.01, partial η^2^ = 0.42), but not across age groups (F _(1, 33)_ = 14.31, *P* = 0.26). The age group by task factor interaction was not significant in each parameter (F _(2, 32)_ = 1.26, *P* = 0.29). Post hoc testing showed Oxy-Hb values during the stepping task were significantly lower than during the calculation task (*P* < 0.01) and the dual-task (*P* < 0.01). The Oxy-Hb values in the time series demonstrated a main effect for the time factor (F _(6, 198)_ = 12.86, *P* < 0.01, partial η^2^ = 0.28). The interactions were significant between time factor and age group (F _(6, 198)_ = 3.29, *P* < 0.01, partial η^2^ = 0.09) and time factor by task factor (F _(12, 396)_ = 8.91, *P* < 0.01, partial η^2^ = 0.21). Examined across the study interval, Oxy-Hb values during the dual-task showed a significant time factor by age factor interaction (F _(6, 198)_ = 4.31, *P* < 0.01, partial η^2^ = 0.21). Post-hoc test results showed that the older group demonstrated higher Oxy-Hb values than the younger group (*P* < 0.01) within the post-task 0-10s period for the dual-task activity. There were no significant time factors by age factor interactions in either the calculation (F _(6, 198)_ = 1.00, *P* = 0.42) or the stepping single-task activities (F _(6, 198)_ = 1.09, *P* = 0.37) (See: Figure 2 and 3).

**Table 2:** The Oxy-Hb value in whole task period

	**Younger group**	**Older group**
Dual-task	0.024 ± 0.056	0.049 ± 0.045
Calculation task	0.036 ± 0.047	0.037 ± 0.030
Stepping task	−0.015 ± 0.038	−0.015 ± 0.015

mean ± S.D.

### Correlation between dual-task performance, Oxy-Hb values and the TMT-B in the older group

**Figure 4:**
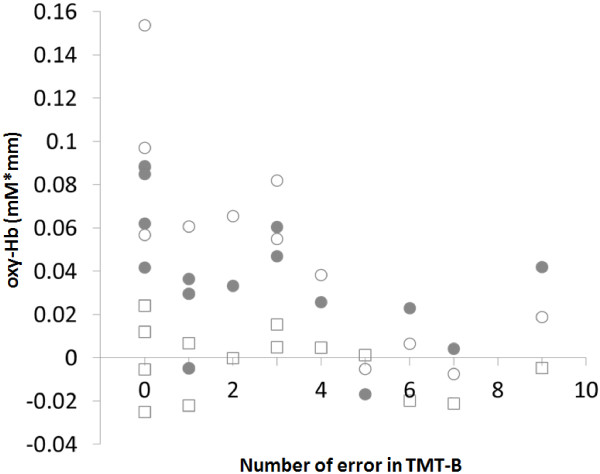
**Correlation between Oxy-Hb values and the TMT-B in the older group.** (○): dual-task, (●): calculation task, (□): stepping task.

There was a significant negative correlation between the Oxy-Hb values during the dual-task and the number of errors in TMT-B (r = −0.63, *P* < 0.05). The number of errors in TMT-B was also negatively correlated with the percentage of correct calculations during the dual-task (r = −0.58, *P* < 0.05). The Oxy-Hb values observed during the calculation task, as well as during the step task, were not significantly correlated with TMT-B scores (calculation task: r = −0.46, *P* = 0.09, stepping task: r = −0.29, *P* = 0.30) (See: Figure 4).

## Discussion

We examined PFC activation by measuring changes in the concentration of Oxy-Hb levels in the PFC using NIRS during a dual-task condition and compared this with measured activation during two different single-tasks (calculation and stepping). The results demonstrated that: 1) Oxy-Hb values were significantly increased during both the calculation single-task and the combined dual-task; 2) during the dual-task, the older adults demonstrated significantly higher Oxy-Hb values than the younger adults, and 3) the increased activation (higher Oxy-Hb values) associated with dual-task performance in the older participants persisted 10 seconds beyond the completion of the task itself. Also, there was a significant negative correlation between Oxy-Hb values during the dual-task and TMT-B scores. There was also a significant negative correlation between the proportion of correct calculations made during the dual-task and TMT-B scores.

Increasing Oxy-Hb values has been demonstrated to be related to increases in cerebral blood volume in response to neuronal activation [12]. Measured Oxy-Hb was significantly increased during both the dual-task and the calculation single-task compared to the stepping single-task. As expected, increased Oxy-Hb was not observed in the PFC during the stepping single-task because this is a simple motor task which does not require activation in the PFC. It is possible that the stepping single-task might increase Oxy-Hb in motor-related areas of the brain. However, this was not measured during our study. Both the calculation single-task and the combined dual-task require PCF activation and our findings support the results reported in previous studies [21,22,43,44]. It has been suggested that actively increasing PFC activation in aging individuals might help to prevent or delay age-related changes to the brain [10,13]. Therefore, a regular schedule of dual-task training for older individuals might mitigate the age-related decline of cognitive function often observed in older adults.

Time series measurements of Oxy-Hb values in both the stepping and calculating single-tasks demonstrated no significant difference between younger and older participants. This suggests that PFC activities in response to either single-task did not differ across age groups. However, significantly different temporal patterns of Oxy-Hb values were observed between the two age groups when performing the combined dual-task. In the older group, Oxy-Hb levels gradually increased and this increase was sustained until the completion of the dual-task. Among younger participants, Oxy-Hb levels increased rapidly at the onset of the dual-task and decreased substantially prior to task completion. Reuter-Lorenz and Cappell [30] have demonstrated overactivation in lateral and inferior PFC during higher levels of task demand in older individuals. Our results support previous reports that dual-task activity is associated with increased brain activation due to higher cognitive load in older participants. The results of this study, which indicated that older participants had higher PFC activation during the dual-task period, correspond with those reported by Reuter-Lorenz and Cappell [30] and suggest that performing a dual-task might impose a higher cognitive load in the older individuals compared with the younger participants. During dual-task performance, the number of steps counted by older participants was significantly decreased compared to the number of steps counted during performance of the stepping single-task. However, among older subjects, neither the number of calculations, nor the proportion performed correctly differed significantly during the dual-task from those observed during single-task performance. From our observations during the dual-task, it appears that older participants may turn their attention to the execution of the calculation task at the expense of the stepping task while young subjects are able to maintain appropriate attention to both calculation and stepping. This finding suggests that older participants required increased PFC activation in order to maintain attention to both the stepping and calculating tasks. It is possible that this requirement for increased PFC activation is related to decreased brain function associated with aging.

The comparison of single-task performance between young and older participant demonstrated a significant different in the proportion of calculations performed correctly, but no significant different in the total number of calculations performed or in the total number of steps. Also, there was no significant difference in task-related Oxy-Hb levels across age groups for each single-task (calculation and stepping). These results suggest that the calculation task was more difficult for the older participants compared with the younger group even though PFC activation was not significantly different across the two age groups during the calculation task. Schroeter [31] indicated that aging is generally associated with decreases in the hemodynamic response within the frontal association cortex during cognitive tasks. However, Reuter-Lorenz and Cappell [30] suggested older adult demonstrated overactivation in PFC during a particularly challenging cognitive task. Our results are in line with those reported by Reuter-Lorenz and Cappell [30], in that older participants had increasing PFC activation during the higher cognitive load imposed by the dual-task. It also seems likely that the calculating/stepping dual-task used in this study was not sufficiently challenging to be affected by the decreased activation observed in older adults exposed to highly complex tasks observed by Callicott et al. [29].

We also analyzed the relationship between brain activity during dual-task performance and participant executive function and attentional function, as measured by the TMT-B. Oxy-Hb values were negatively correlated with the number of errors in TMT-B. Previous studies suggested that PFC volume decreases with age [45] and that cerebral blood volume decreases with dementia [46]. Therefore, the relationship we observed between lower PFC Oxy-Hb values and a greater number of errors on the TMT-B test may reflect a decrease in brain function due to aging-related cognitive decline or incipient dementia. Moreover, our result demonstrated a significant negative correlation between dual-task performance and TMT-B scores among the older study participants. Montero-Odasso et al. [47] have demonstrated that dual-task performance correlates with TMT-B performance. Successful dual-task performance requires attentional function and working memory. Decreases in these functions have been associated with decreased PFC activation in older adults.

There were some limitations in this study. First, because of limitations imposed by the measurement environment, this study used stepping in a chair as a physical activity task. Second, previous study has shown that elderly people with dementia demonstrate reduced brain activation during a cognitive task compared with individuals without dementia [48]. All participants in this study, both older and younger, were cognitively intact so it is not possible to extrapolate these results to populations of older adults with pre-existing changes in cognitive ability. Finally, while our study might suggest that structured dual-task activity facilitates PFC activation in older adults, future study will need to clarify whether dual-task training can lead to facilitated PFC activation in older adults enabling them to maintain cognitive function.

## Conclusions

In conclusion, both younger and older participants showed significantly greater PFC activation during the dual-task compared to either of the tasks in isolation. As a group, older participants demonstrated higher peak Oxy-Hb and longer PFC activation during the dual-task compared with their younger counterparts. This result indicates that dual-task activity imposes a greater cognitive load on older individuals and performing the dual-task more strongly affected changes in brain activation among the older group. A significant negative correlation between PFC hemodynamic activation during dual-task and executive-attentional function in older participants was observed. Therefore, our findings suggest that the increased cognitive load induced by dual-task activity leads to higher PFC activity in older adults and that higher levels of increased activation during the dual-task are associated with a significantly lower error rate on the TMT-B test. It seems possible that, in the therapeutic environment, appropriately designed dual-task training may be a useful tool for the prevention or delay of some aspects of cognitive decline. Further research is warranted to determine how best to design dual-task interventions for vulnerable populations, as well as to assess the value of such interventions on the maintenance of cognitive and functional ability as individuals grow older.

## Abbreviations

PFC: Prefrontal cortex; NIRS: Near-infrared spectroscopy; TMT: Trail-Making Test; TMT-B: Trail-making test part B; Oxy-Hb: Oxygenated hemoglobin; Deoxy-Hb: Deoxygenated hemoglobin; Total-Hb: Total hemoglobin; IGF-I: Insulin-like growth factor-1; ROI: Region of interest; MMSE: Mini-mental state examination.

## Competing interests

The authors declare that they have no competing interests.

## Authors’ contributions

HO was the lead writer of this manuscript and has made study design, acquisition of data, or analysis and interpretation of data. SO had a major role in the conception and design of the study, supervised all aspects of the study's implementation. KS and EBS had assisted the data interpretation and had contributed to the writing of the manuscript. All authors read and approved the final manuscript.
